# Construction of a Prognostic Immune Signature for Squamous-Cell Lung Cancer to Predict Survival

**DOI:** 10.3389/fimmu.2020.01933

**Published:** 2020-09-15

**Authors:** Rui-Lian Chen, Jing-Xu Zhou, Yang Cao, Ling-Ling Sun, Shan Su, Xiao-Jie Deng, Jie-Tao Lin, Zhi-Wei Xiao, Zhuang-Zhong Chen, Si-Yu Wang, Li-Zhu Lin

**Affiliations:** ^1^Integrative Cancer Centre, The First Affiliated Hospital of Guangzhou University of Chinese Medicine, Guangzhou, China; ^2^Department of Oncology, Guangzhou Chest Hospital, Guangzhou, China; ^3^Department of Oncology, Shenzhen People’s Hospital, The Second Clinical Medical College of Jinan University, Shenzhen, China; ^4^Department of Thoracic Surgery, Sun Yat-sen University Cancer Center, Guangzhou, China

**Keywords:** squamous-cell lung cancer, prognostic, immune-related genes, signature, immune cells, mutation profiles

## Abstract

**Background:**

Limited treatment strategies are available for squamous-cell lung cancer (SQLC) patients. Few studies have addressed whether immune-related genes (IRGs) or the tumor immune microenvironment can predict the prognosis for SQLC patients. Our study aimed to construct a signature predict prognosis for SQLC patients based on IRGs.

**Methods:**

We constructed and validated a signature from SQLC patients in The Cancer Genome Atlas (TCGA) using bioinformatics analysis. The underlying mechanisms of the signature were also explored with immune cells and mutation profiles.

**Results:**

A total of 464 eligible SQLC patients from TCGA dataset were enrolled and were randomly divided into the training cohort (*n* = 232) and the testing cohort (*n* = 232). Eight differentially expressed IRGs were identified and applied to construct the immune signature in the training cohort. The signature showed a significant difference in overall survival (OS) between low-risk and high-risk cohorts (*P* < 0.001), with an area under the curve of 0.76. The predictive capability was verified with the testing and total cohorts. Multivariate analysis revealed that the 8-IRG signature served as an independent prognostic factor for OS in SQLC patients. Naive B cells, resting memory CD4 T cells, follicular helper T cells, and M2 macrophages were found to significantly associate with OS. There was no statistical difference in terms of tumor mutational burden between the high-risk and low-risk cohorts.

**Conclusion:**

Our study constructed and validated an 8-IRG signature prognostic model that predicts clinical outcomes for SQLC patients. However, this signature model needs further validation with a larger number of patients.

## Introduction

Lung cancer is the most commonly diagnosed cancer and the first leading cause of cancer-related mortality worldwide, making it a major public health concern (1). In 2018, there were an estimated 2,093,876 new cases, and 1,761,007 deaths from lung cancer worldwide (1). There are two common histological types of non-small cell lung cancer (NSCLC): adenocarcinoma carcinoma, which accounts for 70% of NSCLC cases, and squamous carcinoma, which accounts for 30% of cases. Standard treatments, including chemotherapy, radiotherapy, and surgical resection, have improved the prognosis of early stage squamous-cell lung cancer (SQLC) (2). However, it is difficult to prevent metastasis and recurrence of SQLC, which is considered responsible for most SQLC deaths (3). Platinum-based doublet chemotherapy, the standard therapy for advanced SQLC, only obtained poor efficacy, with a median overall survival (OS) of 12.1 months (4). The utility of targeted drugs had brought significant improvements on OS and the quality of life for advanced NSCLC patients (5, 6). However, driver gene alterations are rarely found in SQLC patients, so the benefit from targeted agents is limited (7). Furthermore, most novel drugs, including pemetrexed and bevacizumab, have been approved in the treatment for lung cancer but not for squamous-cell subtype because of the adverse events (8, 9). Thus, there are limited treatment strategies available for SQLC patients. Checkpoint inhibitors, including anti-cytotoxic T lymphocyte antigen 4 (CTLA4), anti-programmed cell death (PD-1), or anti-programmed cell death-ligand 1 (PD-L1), have brought impressive clinical benefit for various cancer types (10, 11). Due to the remarkable response, pembrolizumab was approved as the first-line treatment for recurrent or metastatic SQLC by the United States Food and Drug Administration and National Medical Products Administration of China (12).

Recent studies have shown that several promising biomarkers might help to select patients who were appropriate candidates for immunotherapy (13, 14). PD-L1 protein expression has been reported to predict the response of checkpoint inhibitors (15, 16). Previous studies have indicated that the tumor mutation burden (TMB) and T-cell infiltration levels were related to the efficacy of immunotherapy (13, 17). However, there is no consensus on the biomarkers that can predict prognosis for SQLC patients. The tumor biology and immune microenvironment were so complicated that a single biomarker may be unable to sufficiently predict the clinical outcomes of immunotherapy.

Several studies have demonstrated that immune signatures played an important role in predicting the prognosis of patients with cancers, such as ovarian cancer, colorectal cancer, and cervical cancer. However, few studies have explored whether immune-related genes (IRGs) could be biomarkers for predicting the prognosis of SQLC. Furthermore, diverse treatment outcomes of PD-1 or PD-L1 inhibitors were observed in SQLC patients (12, 18). Therefore, an immune signature of SQLC based on IRGs is urgently needed to predict clinical outcomes. The aim of the current study was to establish an immune signature that predicts the prognosis of SQLC patients based on IRGs or tumor immune microenvironment (TIME). Furthermore, we explored the relationships of the immune signature and the clinical characteristics, immune cell infiltration, and mutation data. This immune signature may help clinicians to provide more precise immunotherapy for SQLC patients.

## Materials and Methods

### Clinical Samples and Data Acquisition

Transcriptome mRNA-sequencing data and clinical information of SQLC patients were downloaded from The Cancer Genome Atlas (TCGA) data portal^1^. These data contained 49 normal and 502 primary SQLC tissues. The raw count data were downloaded for further analyses. Clinical information was also downloaded and extracted from the Immunology Database and Analysis Portal (ImmPort) database^2^. ImmPort is an important foundation of immunology research, which updates immunology data accurately and timely. This database provides a list of IRGs that are involved in the process of immune activity for cancer researchers (19).

### Differentially Expressed Gene Analysis

To select the IRGs involved in the development of SQLC, differentially expressed genes (DEGs) between tumor and normal samples were identified with the limma package^3^. A differential gene expression analysis was performed with a false discovery rate (FDR) < 0.05 and a log2 fold change > 1 as the cutoff values. A list of IRGs was derived from Immport. We identified differently expressed immune-related genes (DE IRGs) at point intersection between the IRGs list and all DEGs. Functional enrichment analyses were performed to investigate the potential molecular mechanisms of the DE IRGs with gene ontology (GO) and Kyoto Encyclopedia of Genes and Genomes (KEGG) enrichment using DAVID. Terms in GO and KEGG with an FDR < 0.05 were considered significantly enriched.

### Development and Validation of the Immune-Related Signature for SQLC

Squamous-cell lung cancer patients from TCGA data were randomly divided into two cohorts, including the training cohort and the testing cohort. The training cohort was used to identify the prognostic immune-related signature and to develop a prognostic immune-related risk model. The testing cohort was used to validate its prognostic capability. We performed a univariate Cox proportional hazard regression analysis to identify the correlation between DE IRGs and OS in the training cohort. To minimize overfitting and to identify the best gene model, survival-related DE IRGs (*P* < 0.05) were evaluated with a least absolute shrinkage and selection operator (LASSO) (20). The risk score was established with the following formula: risk score = expression gene 1 ^∗^coefficient + expression gene 2 ^∗^coefficient + … + expression gene n ^∗^coefficient (21). The risk score was calculated for each patient in the training and testing cohorts based on this model. SQLC patients were divided into the high- and low-risk groups based on the median cutoff of the risk score. We validated the prognostic ability of the immune-related signature by calculating the area under the curve (AUC) and evaluating the survival difference between the high- and low-risk groups (22).

### TMB Analysis

The mutation data for SQLC patients were obtained from the TCGA data portal, and analyzed with maftools (23). For each patient, the TMB score was calculated as follows: (total mutations/total covered bases) × 10^6^ (24).

### Tumor-Infiltrating Immune Cells

We used gene expression RNA-sequencing data from TCGA to estimate the proportions of 22 types of infiltrating immune cells with the CIBERSORT algorithm following the procedure as previously reported (25).

### Statistical Analysis

Differences among variables were analyzed with independent *t* tests, chi-square tests, non-parametric tests, or ANOVA tests. Univariate cox regression analysis and multivariate cox regression were conducted to assess the prognostic effect of the immune signature and clinical characteristics including gender, age, clinical stage, and TNM stage. Statistical analyses were conducted with SPSS 22.0 and R software, version 3.6.1. The heatmap was generated with R package “pheatmap” and the volcano plot was generated with R package “ggplot2”. A two-sided *P* < 0.05 was considered statistically significant.

## Results

### Clinical Characteristics

A total of 502 SQLC patients were identified in the TCGA cohort. In order to reduce the effect of follow-up time on short term, patients with follow-up time less than 30 days were not included in our study. Thus, a total of 464 patients were enrolled, including 344 (74.1%) male and 120 (25.9%) female patients. These SQLC patients were randomly divided into the training cohort (*n* = 232) and the testing cohort (*n* = 232). No significant difference was observed in terms of the clinical characteristics between these two cohorts. The clinical characteristics of the patients are listed in Supplementary Table S1.

### Identification of DE IRGs

**FIGURE 1:**
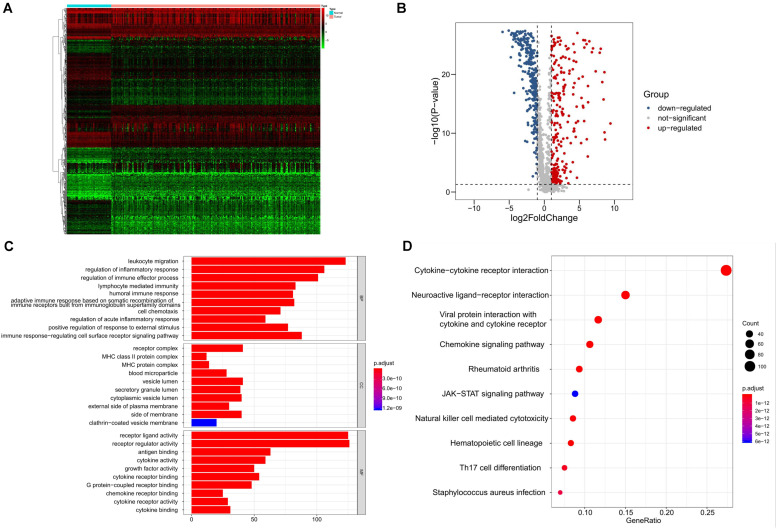
Identification and functional enrichment analyses of differentially expressed immune-related genes in SQLC from training cohort. **(A)** Heatmap of differentially expressed immune-related genes. **(B)** Volcano plot of differentially expressed immune-related genes. **(C)** Gene ontology analysis. **(D)** The top 10 most significant Kyoto Encyclopedia of Genes and Genomes pathways.

We identified 8527 DEGs for SQLC, including 5803 up-regulated and 2724 down-regulated genes (Supplementary Figure S1). We extracted 587 DE IRGs from the set of DEGs, comprising 287 up-regulated and 300 down-regulated genes (Figures 1A,B). Gene functional enrichment analysis indicated that these genes were significantly enriched in important inflammatory pathways, including leukocyte migration, regulation of inflammatory response, regulation of immune effector process, and lymphocyte-mediated immunity (Figure 1C). KEGG pathway analysis highlighted the six ranked pathways that were enriched among the DE IRGs: “cytokine–cytokine receptor interaction”, “neuroactive ligand–receptor interaction”, “viral protein interaction with cytokine and cytokine receptor”, “chemokine signaling pathway”, “rheumatoid arthritis”, and “JAK-STAT signaling pathway” (Figure 1D).

### Construction of Immune-Related Risk Signatures in SQLC

**FIGURE 2:**
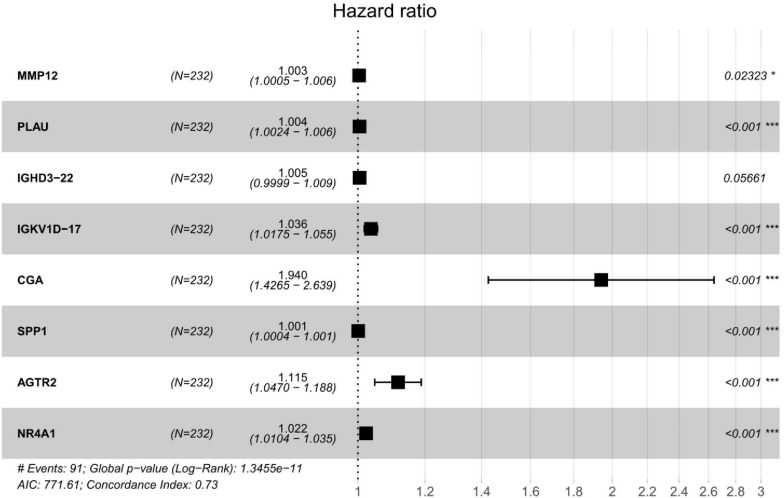
Forest plot of the multivariable Cox model of each gene in 8-IRG risk signature.

**FIGURE 3:**
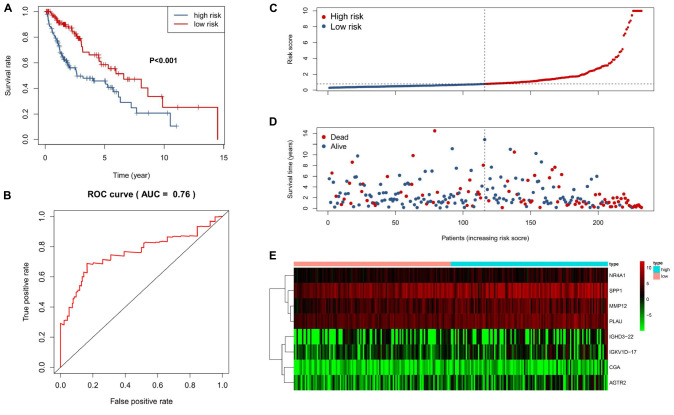
Construction of an 8-IRG signature in the training cohort. **(A)** Kaplan-Meier curve analysis of overall survival of SQLC patients in high- and low-risk groups. **(B)** ROC curves analysis of 1 year. Risk score distribution **(C)**, survival status **(D)**, and heatmap of expression profiles **(E)** for patients in high- and low-risk groups by the 8-IRG signature.

We performed a univariate Cox regression analysis to explore the association between OS and these 587 DE IRGs identified above. A total of 32 DE IRGs were significantly associated with the OS of SQLC patients in the training cohort (*P* < 0.05). LASSO analysis was performed with these 32 survival-associated IRGs in order to minimize overfitting. Eight DE IRGs were utilized to construct the immune signature (Figure 2). The prognostic model was established with the linear combination of the expression levels of the 8-IRGs weighted by their relative coefficient in multivariate Cox regression analysis as follows: risk score = (*MMP12* × 0.00332) + (*PLAU* × 0.00434) + (*IGHD3-22* × 0.00460) + (*IGKV1D-17* × 0.03535) + (*CGA* × 0.66283) + (*SPP1* × 0.00072) + (*AGTR2*× 0.10901) + (*NR4A1* × 0.02224) (Supplementary Table S2). We calculated risk scores for each patient in the training cohort based on the expression of the eight IRGs and their relative coefficient. A total of 232 patients in the training group were divided into a high-risk cohort (*n* = 166) and a low-risk cohort (*n* = 166) based on their median risk score. A significant difference in OS was observed between the high-risk and low-risk cohorts [median OS, 4.56 vs 7.40 years; hazard ratio (HR), 2.21; 95% CI, 1.44–3.41, *P* < 0.001] (Figure 3A). The AUC for the 8-IRG signature was 0.76 at 1 year for OS (Figure 3B). The distribution of the risk score and survival status and the expression of 8-IRGs in the training cohort were presented in Figures 3C–E.

### Evaluating the Predictive Value of the 8-IRG Signature

**FIGURE 4:**
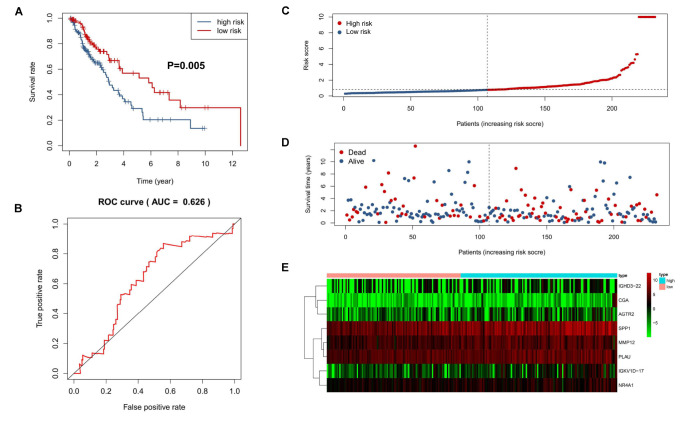
Validation of an 8-IRG signature in the validating cohort. **(A)** Kaplan-Meier curve analysis of overall survival of SQLC patients in high- and low-risk groups. **(B)** ROC curves analysis of 1 year. Risk score distribution **(C)**, survival status **(D)**, and heatmap of expression profiles **(E)** for patients in high- and low-risk groups by the 8-IRG signature.

The predictive capability of the 8-IRG signature was verified with the testing cohort and the total cohort. As previously described, there were 125 high-risk and 107 low-risk patients in the testing cohort. The patients in the high-risk cohort had a significant shorter median OS than those in the low-risk cohort (median OS, 3.93 vs 6.47 years; HR, 1.84; 95% CI, 1.20–2.84; *P* = 0.005; Figure 4A). The AUC of 1 year was 0.63 (Figure 4B). The distribution of the risk score, survival status, and the expression of 8-IRGs in the testing cohort are shown in Figures 4C–E. Similarly, SQLC patients in the total cohort were divided into low-risk (*n* = 223) and high-risk (*n* = 241) groups. The median OS in the high-risk cohort was inferior than that of the low-risk cohort (median OS, 4.34 vs 7.00 years; HR, 2.04; 95% CI, 1.50 to 2.76, *P* < 0.001; Supplementary Figure S2a). The AUC of 1 year in the total cohort was 0.69 (Supplementary Figure S2b). The distribution of the risk score, survival status, and the expression of 8-IRGs in the total cohort are presented in Supplementary Figures S2c–e.

### Association Between the Immune-Related Risk Signature and the Clinical Outcome

A univariate Cox regression model was conducted to explore the association between clinical characteristics, OS, and the 8-IRG risk signature in the total SQLC cohort (Table 1). The immune-related risk signature could independently predict OS in the total cohort (HR, 1.60; 95% CI, 1.17–2.19, *P* = 0.003). Multivariate Cox regression analysis suggested that the immune-related risk signature could act as an independent prognosis predictor for OS (HR, 1.94; 95% CI, 1.38–2.72, *P* < 0.001). The relationships between the immune signature and clinical characteristics were also explored. No significant difference of risk scores was found in terms of age, gender, clinical stage, T stage, and N stage (Supplementary Figure S3).

**TABLE 1:** Univariate and multivariate Cox regression analysis of SQLC.

Variables	Univariate analysis		Multivariate analysis	
	Hazard ratio (95% CI)	*P* value	Hazard ratio (95% CI)	*P* value
Age (≤65 vs. >65)	1.237 (0.908–1.686)	0.178	1.285 (0.932–1.771)	0.126
Gender (male vs. female)	0.899 (0.637–1.268)	0.544	0.800(0.562–1.138)	0.215
**Tumor stage**				
T1	1		1	
T2	1.274 (0.859–1.890)	0.229	1.302(0.862–1.969)	0.210
T3	1.972 (1.204–3.231)	0.007	2.245 (1.093–4.612)	0.028
T4	2.491 (1.308–4.744)	0.005	3.250 (1.288–8.196)	0.013
**N stage**				
N0	1		1	
N1	1.205 (0.862–1.684)	0.275	1.361 (0.780–2.374)	0.278
N2	1.405 (0.862–2.292)	0.173	1.521 (0.622–3.722)	0.358
N3	2.987 (0.733–12.174)	0.127	5.574 (1.005–30.929)	0.049
**Clinical stage**				
I	1		1	
II	1.235 (0.871–1.750)	0.236	0.918 (0.507–1.661)	0.777
III	1.784 (1.229–2.591)	0.002	0.874 (0.330–2.315)	0.787
IV	2.251 (0.707–7.166)	0.170	1.579 (0.419–5.959)	0.500
8-IRG risk score (low vs high)	1.600 (1.168–2.193)	0.003	1.937 (1.382–2.715)	<0.001

*HR, hazard ratio; CI, confidence interval; IRG, immune-related genes.*

### TIME Changing and the Immune-Related Risk Signature

**FIGURE 5:**
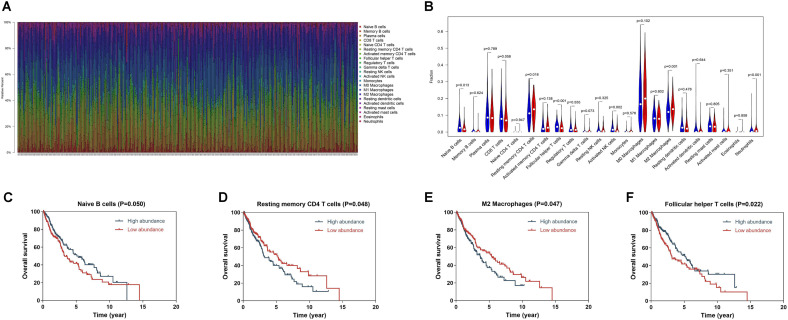
**(A)** Bar chart of the relative proportion of the 22 immune cells in each SQLC sample. **(B)** The association of immune cells infiltration and the immune-related risk signature in SQLC. A red violin and a blue violin represent the 8-IRG signature high-risk and low-risk groups. The white points inside the violin represent median values. **(C–F)** The association of immune cells infiltration and OS in TCGA SQLC dataset. **(C)** Naïve B cells; **(D)** Resting memory CD4 T cells; **(E)** M2 macrophages; **(F)** Follicular helper T cells.

We applied RNA-sequencing data to assess the relative proportion of the 22 immune cells in each SQLC sample with CIBERSORT (Figure 5A). The abundances of the immune cell types in the 8-IRG signature low- and high-risk cohorts are presented in Supplementary Table S4. Among the 22 immune cell types, the proportions of follicular helper T cells, naïve B cells, and activated NK cells were low in the 8-IRG signature high-risk group. The abundances of resting memory CD4 T cells, M2 macrophages, and neutrophils were high in the 8-IRG signature high-risk group (Figure 5B). The proportions of naïve B cells, resting memory CD4 T cells, follicular helper T cells, and M2 macrophages were significantly associated with OS. For the 8-IRG signature in the low-risk cohort, the abundance level of resting memory CD4 T cells and M2 macrophages was low and showed a significant association with superior OS, whereas the abundance levels of naïve B cells and follicular helper T cells were high and were associated with inferior OS (Figures 5C–F).

### Tumor Mutation Profile and the Immune-Related Risk Signature

**FIGURE 6:**
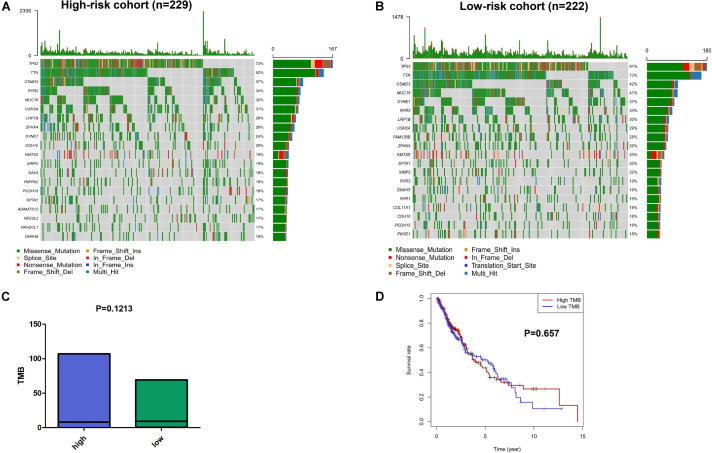
The mutation profiles and TMB among low-risk and high-risk groups. Mutation profile of low-risk **(A)** and high-risk **(B)** groups. **(C)** The relationship between the immune-related risk signature and TMB. **(D)** The association of TMB and OS in TCGA SQLC dataset.

We explored the relationship between the mutation profile and the immune-related risk signature in TCGA SQLC patients with available somatic mutation data. The 30 ranked, mutated genes in the low-risk and high-risk cohorts are illustrated in Figures 6A,B. The top 10 mutated genes in SQLC patients were *TP53*, *TIN*, *CSMD3*, *MUC16*, *RYR2*, *SYNE1*, *USH2A*, *LRP1B*, *ZFHX4*, and *FAM135B*. There were no statistical differences in terms of TMB between the high-risk and low-risk cohorts (*P* = 0.121; Figure 6C). No significant difference in OS was found in the high- or low-TMB cohorts (*P* = 0.657; Figure 6D).

## Discussion

Several clinical trials have shown that checkpoint inhibitors were superior to chemotherapy for SQLC patients (12, 18). However, SQLC patients have shown limited improved clinical outcomes from immunotherapy. Thus, it is important to identify and develop potential biomarkers for predicting prognosis in SQLC patients treated with immunotherapy.

Early studies have demonstrated that PD-L1 expression, T-cell receptor clonality, TMB, and T-cell infiltration levels may be associated with the clinical response to immunotherapy (13, 17). However, due to the complexity of tumor biology and the immune microenvironment, a single biomarker could not be sufficiently predictive of clinical outcomes to immunotherapy (26). It may be necessary to apply the integration genomics and transcriptomic to improve the accuracy of predictions. Furthermore, as the TIME served as a critical role in tumor progression, it is important to explore an immune-related model to predict the prognosis of SQLC patients and identify patients who would obtain clinical benefit from immunotherapy.

To the best of our knowledge, it was the first immune-related signature prognostic model for SQLC patients based on RNA-sequencing data. In our study, we firstly presented the gene mutation profiles and the relative proportion of 22 immune cells of SQLC from TCGA dataset. Besides, the relationships between TMB, proportion of immune cells, and SQLC prognosis were firstly systematic exploration in our article.

Our study established and validated an immune-related risk signature model for SQLC from TCGA dataset. A total of eight DE IRGs with prognostic value were included in the signature. Among these genes, six (*MMP12*, *PLAU*, *IGHD3-22*, *IGKV1D-17*, *CGA*, and *SPP1*) were up-regulated in SQLC tissues compared with normal samples, while two (*AGTR2* and *NR4A1*) were down-regulated. *PLAU* and *MMP12* have been reported to be associated with aberrant regulation of gene function and poor prognosis for lung carcinoma (27–30). *SPP1* has been reported as an independent risk biomarker prognostic evaluation of patients with lung adenocarcinoma (31). *NR4A1* has been considered as a member of the orphan nuclear receptor superfamily of transcription factors (32). In our study, *NR4A1* was down-regulated in the SQLC tissues compared with the normal tissues. However, *NR4A1* has been reported to be overexpressed in multiple types of carcinomas in previous reports, and play a critical role in survival or cell proliferation in cervical, lymphoma, pancreatic, lung, and colon cancer cells. *NR4A1* has been found to be involved in promoting cancer invasion and metastasis (33–35). A previous research showed that *AGTR2* was under-expressed in lung adenocarcinoma and played a role in the pathology of adenocarcinoma (36). *CGA* gene was identified as a new estrogen receptor a (ERa) responsive gene in human breast cancer cells and a member of a novel dysregulated pathway in prostate cancer (37–39). *IGKV1-17* gene was reported to be rarely expressed by normal cells and play a critical role in the development of SLE-nephritis (40). *IGHD* gene served as suppressor genes in the recurrence of triple-negative breast cancer (41). Although the role of the remaining *CGA*, *IGHD3-22*, and *IGKV1D-17* genes in lung cancer patients has not been previously reported, they might play an important role as potential biomarkers.

We found that four IRGs encoded cytokines or cytokine receptors, including *CGA*, *SPP1*, *AGTR2*, and *NR4A1* genes. Cytokines and cytokine receptors have been reported to modulate the tumor microenvironment and promote the development of cancer, which may contribute to disease progression and a worse prognosis for SQLC patients in the high-risk group (42–44). Significant differences in OS were found between patients with high-risk and low-risk scores. Furthermore, our signature was significantly associated with the prognosis of SQLC patients in the training, testing, and total cohorts. Our 8-IRG signature has acted as an independent prognostic factor in OS for SQLC patients in both the univariate and the multivariate Cox regression analyses. These results demonstrated that the signature might be a useful tool for predicting prognosis.

Our signature had also shown relationships with immune cells. CIBERSORT was applied to assess the relative abundances of 22 immune cells types in each SQLC sample. Our study showed that the proportion of resting memory CD4 T cells, M2 macrophages, and neutrophils were positively correlated with 8-IRG risk score, and the proportion of follicular helper T cells, naïve B cells, and activated NK cells were negatively associated with the 8-IRG risk score. Furthermore, high abundance levels of resting memory CD4 T cells and M2 macrophages were found in the high-risk cohort, which was associated with poorer OS. Low abundance levels of naïve B cells and follicular helper T cells were found in the high-risk cohort, which was associated with better OS. High proportion of M2 macrophage was reported to be correlated with a poor response to immunotherapy (45). These results may contribute to the poor prognosis in the high-risk cohort.

We also performed gene mutation analysis to explore the possible mechanisms of the 8-IRG signature in the high- and low-risk groups. However, there was no significant difference in TMB between the 8-IRG signature high-risk group and low-risk group. Furthermore, our study showed that TMB was not associated with OS, which was not consistent with the results of previously reported studies (17, 46). However, a recent study showed that there was no significant correlation between TMB and the prognosis of lung cancer patients treated with pembrolizumab (26). According to the NCCN guideline for NSCLC patients, TMB is an evolving biomarker that may be helpful to select patients for immunotherapy, but there is no consensus on how to measure TMB in clinical practices (14).

Despite these promising results, there were several limitations in our study. First, the immune-related signature model was established and validated with gene profiles from the public dataset. Second, the proportion of Asian SQLC patients was small in the TCGA cohort. Thus, it is still unclear whether this signature model will be effective for Asian SQLC patients. Further studies should incorporate with a larger number of SQLC patients from Asia and the clinical practice.

## Conclusion

Our study constructed and validated an 8-IRG signature prognostic model to predict clinical outcomes for SQLC patients, which may provide a deeper understanding of immunotherapy. However, this signature model for SQLC needs further validation with a larger number of patients.

## Data Availability Statement

The gene expression RNA-sequencing data, mutation data, and clinical information of SQLC patients in our study were downloaded from the TCGA data portal (https://portal.gdc.cancer.gov). The comprehensive list of IRGs was obtained from the ImmPort database (https://immport.niaid.nih.gov).

## Author Contributions

R-LC made contributions to conception, design, and data analysis and wrote the manuscript. SS, X-JD, and L-LS analyzed the data. J-TL, Z-WX, Z-ZC, and S-YW gave suggestions on study design, discussed, and interpreted the data. J-XZ, YC, and L-ZL designed and supervised the study. All authors read and approved the final version of the manuscript.

## Conflict of Interest

The authors declare that the research was conducted in the absence of any commercial or financial relationships that could be construed as a potential conflict of interest.

## Footnotes

1 https://cancergenome.nih.gov

2 https://immport.niaid.nih.gov

3 https://Bioconductor.org/packages/limma
