# Supplementary material for: Construction of a Prognostic Immune Signature for Squamous-Cell Lung Cancer to Predict Survival
Source: Front Immunol. 2020 Sep 15;11:1933. doi: 10.3389/fimmu.2020.01933 (PMC7533590; doi:10.3389/fimmu.2020.01933)
Supplement: Supplementary file 1 [file Table_1.docx]

Table S1. Clinical characteristics in the training and testing cohorts.

| Characteristic | Group | Total cohort(n=464) | Training cohort (n=232) | Testing cohort (n=232) | P value |
| --- | --- | --- | --- | --- | --- |
| Age (years) | ≤65 | 181 | 97 | 84 | 0.216 |
|  | ＞65 | 283 | 135 | 148 |  |
| Gender | Male | 344 | 170 | 174 | 0,672 |
|  | Female | 120 | 62 | 58 |  |
| Clinical stage | I | 221 | 114 | 107 | 0.717 |
|  | II | 153 | 74 | 79 |  |
|  | III | 80 | 39 | 41 |  |
|  | IV | 6 | 2 | 4 |  |
|  | NA | 4 | 3 | 1 |  |
| T stage | T1 | 104 | 51 | 53 | 0.962 |
|  | T2 | 272 | 135 | 137 |  |
|  | T3 | 68 | 36 | 32 |  |
|  | T4 | 20 | 10 | 10 |  |
| N stage | N0 | 293 | 156 | 137 | 0,101 |
|  | N1 | 123 | 50 | 73 |  |
|  | N2 | 39 | 21 | 18 |  |
|  | N3 | 4 | 3 | 1 |  |
|  | Nx | 4 | 1 | 3 |  |
| M stage | M0 | 384 | 190 | 194 | 0.550 |
|  | M1 | 6 | 2 | 4 |  |
|  | Mx | 74 | 40 | 34 |  |

NA, not available; Nx, unknown N stage; Mx, unknown M stage.

Table S2. Coefficients and multivariable Cox model results of each gene in 8-IRG risk signature.

| Gene | Log FC | Regulation | Coefficient | HR | P value |
| --- | --- | --- | --- | --- | --- |
| MMP12 | 5.785753 | Up | 0.00332 | 1.00333 | 0.02323 |
| PLAU | 2.838077 | Up | 0.00434 | 1.00436 | 0.00001 |
| IGHD3-22 | 1.096164 | Up | 0.00460 | 1.00462 | 0.05661 |
| IGKV1D-17 | 1.897065 | Up | 0.03535 | 1.03599 | 0.00012 |
| CGA | 6.81337 | Up | 0.66283 | 1.94028 | 0.00002 |
| SPP1 | 4.521234 | Up | 0.00072 | 1.00073 | 0.00004 |
| AGTR2 | -3.29743 | Down | 0.10901 | 1.11518 | 0.00071 |
| NR4A1 | -2.73576 | Down | 0.02224 | 1.02249 | 0.00024 |

HR, hazard ratio; FC, fold change.

Table S3. Gene list and immune category for 8-IRG signature.

| Gene | ID | Name | Category |
| --- | --- | --- | --- |
| MMP12 | 4321 | matrix metallopeptidase 12 | Antimicrobials |
| PLAU | [5328](http://www.ncbi.nlm.nih.gov/gene/5328) | plasminogen activator, urokinase | Antimicrobials |
| IGHD3-22 | [28497](http://www.ncbi.nlm.nih.gov/gene/28497) | immunoglobulin heavy diversity 3-22 | BCR Signaling Pathway |
| IGKV1D-17 | 28900 | immunoglobulin kappa variable 1D-17 | BCR Signaling Pathway |
| CGA | [1081](http://www.ncbi.nlm.nih.gov/gene/1081) | glycoprotein hormones, alpha polypeptide | Cytokine |
| SPP1 | [6696](http://www.ncbi.nlm.nih.gov/gene/6696) | secreted phosphoprotein 1 | Cytokine |
| AGTR2 | [186](http://www.ncbi.nlm.nih.gov/gene/186) | angiotensin II receptor type 2 | Cytokine Receptors |
| NR4A1 | 3164 | angiotensin II receptor type 2 | Cytokine Receptors |

Table S4, Immune cell type abundance between the 8-IRG signature low-risk cohort and high-risk cohort in the total set.

|  | Abundance | |  |
| --- | --- | --- | --- |
| Immune cell type | Low-risk (n=200) | High-risk (n=224) | P value |
| Naïve B cells | 0.0248 | 0.015689 | 0.0.13 |
| Memory B cells | 0 | 0 | 0.624 |
| Plasma cells | 0.085699 | 0.082714 | 0.789 |
| CD8 T cells | 0.0761 | 0.069064 | 0.058 |
| Naïve CD4 T cells | 0 | 0 | 0.947 |
| Resting memory CD4 T cells | 0.111246 | 0.133708 | 0.018 |
| Activated memory CD4 T cells | 0.018122 | 0.025096 | 0.138 |
| Follicular helper T cells | 0.030599 | 0.022601 | <0.001 |
| Regulatory T cells | 0.011266 | 0.01212 | 0.555 |
| Gamma delta T cells | 0 | 0 | 0.073 |
| Resting NK cells | 0.0019 | 0.004804 | 0.325 |
| Activated NK cells | 0.013162 | 0.003624 | 0.02 |
| Monocytes | 0 | 0 | 0.576 |
| M0 Macrophages | 0.165724 | 0.198445 | 0.102 |
| M1 Macrophages | 0.080546 | 0.078348 | 0.802 |
| M2 Macrophages | 0.119569 | 0.135883 | 0.001 |
| Resting dendritic cells | 0.025778 | 0.019491 | 0.478 |
| Activated dendritic cells | 0 | 0.000152 | 0.644 |
| Resting mast cells | 0.034229 | 0.030695 | 0.805 |
| Activated mast cells | 0 | 0 | 0.351 |
| Eosinophils | 0 | 0 | 0.858 |
| Neutrophils | 0.000212 | 0.005022 | <0.001 |

*The data are presented as median.
