# Supplementary material for: Construction of a Prognostic Immune Signature for Squamous-Cell Lung Cancer to Predict Survival
Source: Front Immunol. 2020 Sep 15;11:1933. doi: 10.3389/fimmu.2020.01933 (PMC7533590; doi:10.3389/fimmu.2020.01933)

Supplementary materials

Figure S1. Identification of differentially expressed genes in SQLC from TCGA dataset. (A)The heatmap of differentially expressed genes. (B) Volcano plot of differentially expressed genes.


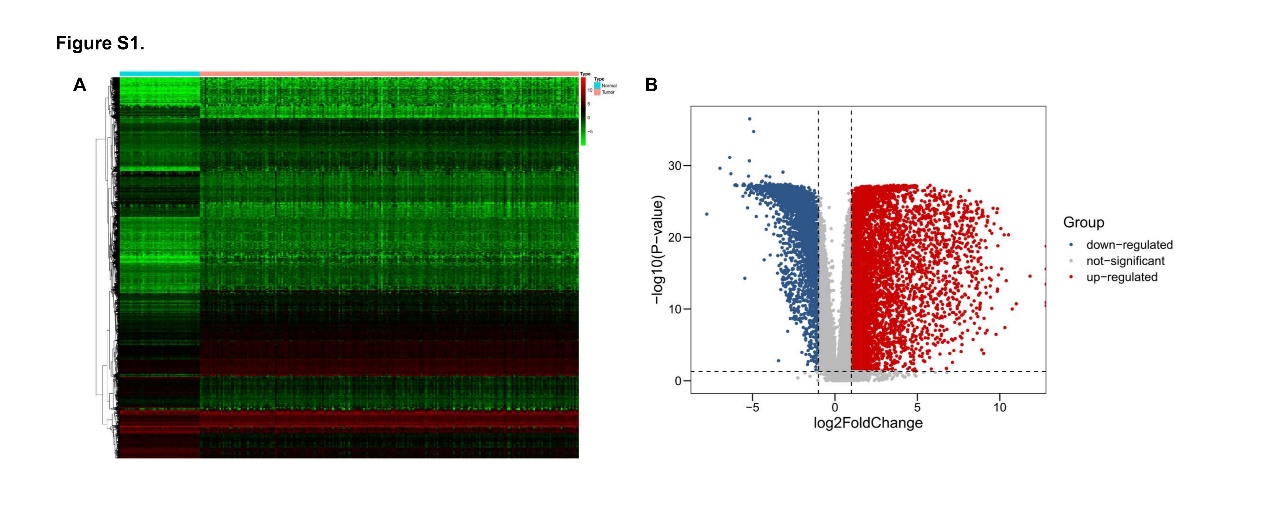


Figure S2. Validation of an 8-IRG signature in the total cohort. (a) Kaplan-Meier curve analysis of overall survival of SQLC patients in high- and low-risk groups. (b) ROC curves analysis of one year. Risk score distribution (c), survival status (d), and heatmap of expression profiles (e) for patients in high- and low-risk groups by the 8-IRG signature.


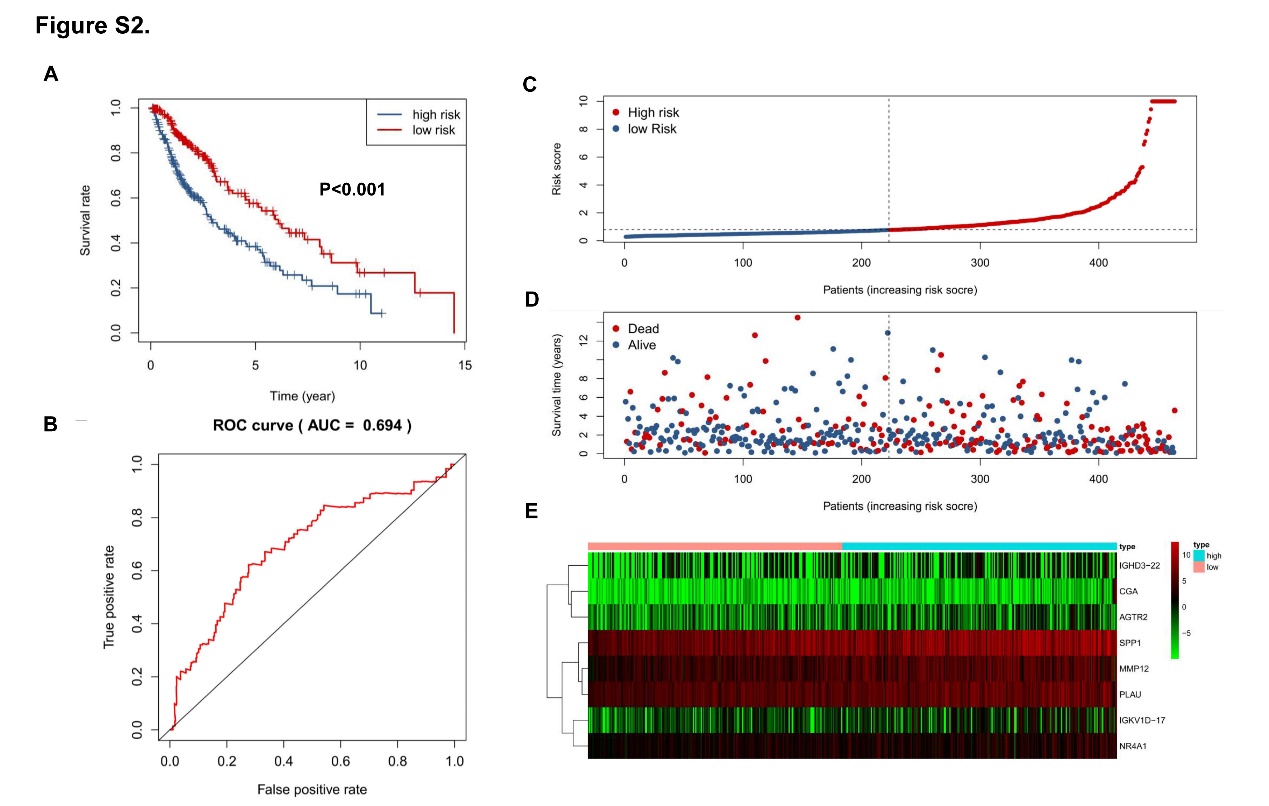


Figure S3. The relationships between the risk scores based on 8-IRG signature and (A) age; (B) gender; (C) clinical stage; (D) T stage; (E) N stage.


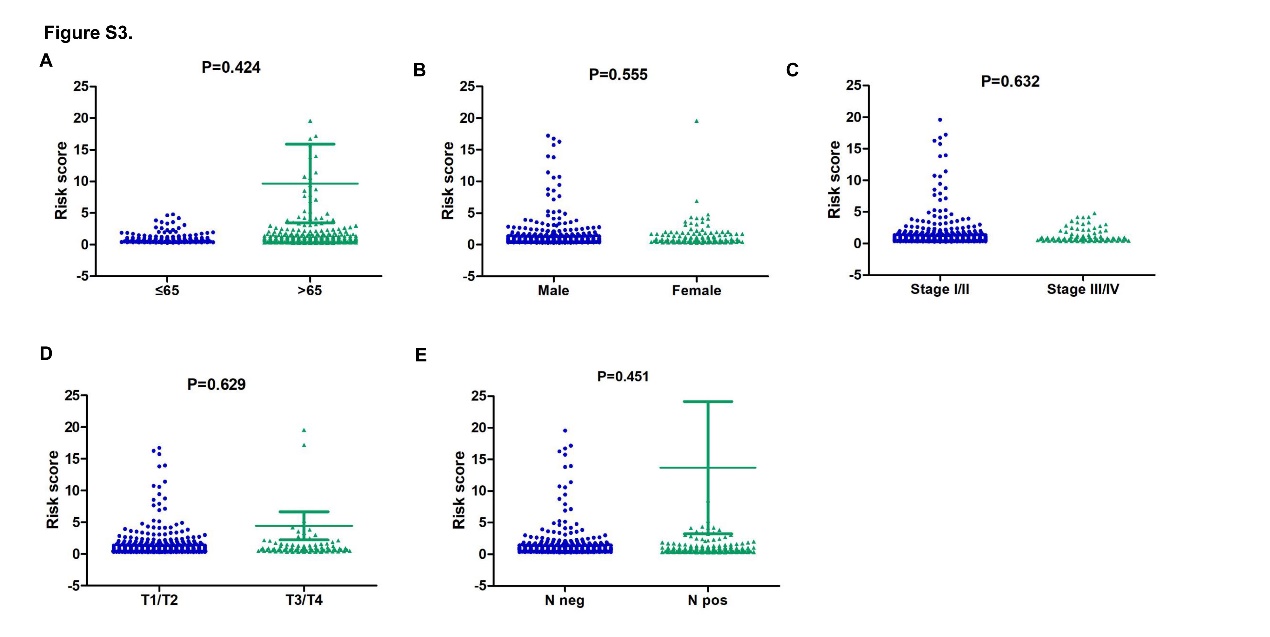

Supplement: Supplementary file 2 [file Table_2.docx]
